# Neuro-Sjögren: Peripheral Neuropathy With Limb Weakness in Sjögren's Syndrome

**DOI:** 10.3389/fimmu.2019.01600

**Published:** 2019-07-11

**Authors:** Tabea Seeliger, Nils K. Prenzler, Stefan Gingele, Benjamin Seeliger, Sonja Körner, Thea Thiele, Lena Bönig, Kurt-Wolfram Sühs, Torsten Witte, Martin Stangel, Thomas Skripuletz

**Affiliations:** ^1^Department of Neurology, Hanover Medical School, Hanover, Germany; ^2^Department of Otolaryngology, Hanover Medical School, Hanover, Germany; ^3^Department of Respiratory Medicine, Hanover Medical School, Hanover, Germany; ^4^Department of Clinical Immunology and Rheumatology, Hanover Medical School, Hanover, Germany

**Keywords:** Sjögren's syndrome, Neuro-Sjögren, neuropathy, chronic inflammatory demyelinating polyneuropathy, motoneuron disease

## Abstract

**Objective:** Sjögren's syndrome is a heterogeneous inflammatory disorder frequently involving peripheral nerves with a wide spectrum of sensory modalities and distribution patterns. The objective of this cross-sectional study was to determine characteristics of Sjögren's syndrome as a cause for severe neuropathy with limb weakness.

**Methods:** One hundred and eighty four patients with polyneuropathy associated with limb weakness underwent routine diagnostics including investigations for Sjögren's syndrome. Forty-four patients with Sjögren's syndrome (ACR-EULAR classification criteria) and severe neuropathy were identified.

**Results:** Sjögren's syndrome was found at a median age of 63 years and the gender distribution showed a balanced female-male ratio of 1:1. Anti-SSA(Ro) antibodies were detected in 48% while seronegative patients were diagnosed with Sjögren's syndrome based on sialadenitis on minor salivary gland biopsy with a focus score ≥1. The majority of patients (93%) were diagnosed with Sjögren's syndrome after neurological symptoms appeared. Limbs were symmetrically involved in 84% of patients (57% tetraparesis, 27% paraparesis). Sensory function was not affected in 11% of patients indicating that Sjögren's syndrome associated neuropathy can present as a pure motor syndrome. Electrophysiological measurements did not reveal pathognomonic findings (23% demyelinating pattern, 36% axonal pattern, 41% both demyelinating and axonal damage signs). More than half of our patients fulfilled the European Federation of Neurological Societies (EFNS) diagnostic criteria for CIDP indicating that distinction between Neuro-Sjögren and other causes of neuropathy such as CIDP is challenging.

**Interpretation:** Our findings show that severe neuropathy with limb weakness is often associated with Sjögren's syndrome. This is of great importance in identifying and understanding the causes of immune mediated polyneuropathy.

## Introduction

Sjögren's syndrome has been described as an inflammatory disease of salivary and lacrimal glands characterized by typical sicca symptoms of dry mouth and eyes and lymphocytic infiltration of glandular tissues (1). Extraglandular manifestations are common and include inflammation of joints, skin, kidney, heart, lung, and intestines (2). Inflammation of the nervous system represents another complication of Sjögren's syndrome. Involvement of peripheral nerves predominantly presents with sensory neuropathy and a wide spectrum of sensory modalities and distribution patterns (3). Motor impairment due to neuropathy has not been widely recognized until now in patients with Sjögren's syndrome (3, 4). However, this severe complication of Sjögren's syndrome with fulminant development of limb weakness but favorable outcome after immunosuppressive therapy was described in several case reports (5–9). Since the prevalence of peripheral neuropathy associated with Sjögren's syndrome increases with age (10), underestimation of paralysis as a neurologic complication of Sjögren's syndrome is a great risk. Patients presenting with limb weakness as a main complaint of polyneuropathy are predominantly treated in neurological departments, where a potentially causal Sjögren's syndrome may not be routinely investigated. Since Sjögren's syndrome is usually diagnosed in immunological departments, rheumatologists are more familiar with this rare autoimmune disease than neurologists. In order to define this entity in detail, we assessed the clinical picture of Sjögren's syndrome patients with neuropathy and motor dysfunction to facilitate diagnostic approaches and treatment eligibility.

## Methods

### Patient Selection and Disability Scoring

Analyses were performed from patients treated as inpatients at the Department of Neurology of the Hannover Medical School between 10/2015 and 02/2018. Patients were included when there were symptoms and signs of a severe progressive symmetric or asymmetric peripheral neuropathy. This was defined by obligatory objective limb weakness and electrophysiological impairment (demyelination and/or axonal damage) of more than one peripheral nerve. Patients with isolated sensory deficits and patients with confirmed or highly suspected additional central nervous system manifestation were excluded from the analysis. Patients with suggested infectious, toxic, metabolic, amyloid, or paraneoplastic neuropathy were excluded. In case of paraproteinemia investigation for hematologic malignancy was performed. Overall, 184 patients with severe polyneuropathy associated with limb weakness underwent routine diagnostics including investigation for Sjögren's syndrome as a possible cause, and 44 of these 184 patients fulfilled the ACR-EULAR-classification criteria for Sjögren's syndrome. Patients with a marked bilateral difference of upper or lower extremities in strength (two or more Medical Research Council grades) or sensory function were defined as having an asymmetric form of peripheral neuropathy. Patients' disability was graded by the overall disability sum score [ODSS], ranging from 0 (no signs of disability) to 12 (maximum disability comprising severe symptoms in both arms preventing all purposeful movements and implying restriction to wheelchair or bed most of the day) (11, 12).

### Sjögren's Syndrome Classification Criteria

The ACR-EULAR classification criteria for Sjögren's syndrome (13) were reappraised for every patient. As suggested, patients were only eligible to classification if they stated at least one symptom of ocular or oral dryness (defined as recurrent sensation of dry eyes for at least 3 months, use of tear substitute >3 times daily, daily experience of a dry mouth and the need of liquids while swallowing). Classification of Sjögren's syndrome was performed when summing up the weights of the following items resulted in a score of ≥4: labial minor salivary gland biopsy with focal lymphocytic sialadenitis and focus score ≥1 (3, 14), positive anti-SSA(Ro) antibodies (3), Schirmer test ≤ 5 mm/5 min on at least one eye (1), ocular staining score ≥5 on at least one eye (1), and unstimulated whole saliva flow rate ≤ 0.1 ml/min (1). According to the recommendations, a history of head and neck radiation treatment, active hepatitis C infection, acquired immunodeficiency syndrome, sarcoidosis, amyloidosis, graft vs. host disease, and IgG4-related diseases were ruled out.

**Figure 1:**
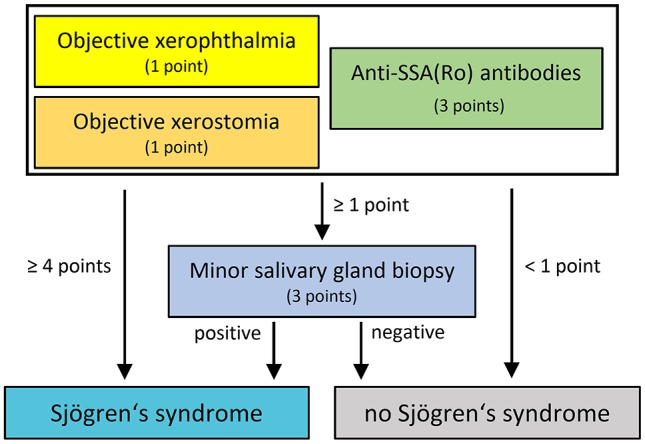
Diagnostic approach for evaluation of Sjögren's syndrome in patients with polyneuropathy.

All patients underwent laboratory testing for anti-SSA(Ro)-antibodies. Tear production was investigated by the Schirmer test, while saliva production was examined by the Saxon test (15). Minor salivary gland biopsy was performed in 41/44 patients. The other 3 patients refused minor salivary gland biopsy. The diagnostic work-up is illustrated in Figure 1.

### Cerebrospinal Fluid (CSF) and Laboratory Data

CSF and serum were analyzed by routine methods (4, 16). CSF leukocytes were counted manually with a Fuchs-Rosenthal chamber and >4 cells/μl were designated as elevated. CSF total protein (cut off = 500 mg/L) was determined by a Bradford dye-binding procedure. Albumin, IgG, IgA, and IgM were examined in serum and CSF in the same assay by kinetic nephelometry (Beckman Coulter IMMAGE). The blood-CSF barrier dysfunction was defined as CSF/serum albumin quotients (Qalbumin) higher than the age-adjusted upper reference limit of Qalbumin which was calculated [Qalbumin = (age in years/15) + 4] (17). CSF specific oligoclonal bands were determined by isoelectric focusing in polyacrylamide gels with consecutive silver staining. Anti-Ro antibodies were measured by EliA (ThermoScientific), Rheumatoid Factor was measured by laser nephelometry (Siemens BN ProSpec), and α-Fodrin IgA and IgG were measured by enzyme-linked immunosorbent assay (ELISA, Aesku.Diagnostics).

### Nerve Conduction Studies

Standardized electrophysiological diagnostics were performed by using superficial stimulators and recording electrodes according to the recommendations of the International Federation of Clinical Neurophysiology (18). Nerve conduction studies were performed on median, ulnar, peroneal, tibial, and sural nerves depending on the clinical manifestation. Evident polyneuropathy was classified as primarily axonal, primarily demyelinating, or mixed axonal and demyelinating. Classification was assigned depending on signs of the respective damage mechanism in the majority of nerves (Supplementary Table 1). The first available neurographic analyses were considered in order to identify the primary damage mechanism. In addition, diagnostic criteria for chronic inflammatory demyelinating polyneuropathy (CIDP) were reappraised for every patient (19).

### Statistical Analysis and Data Collection

Descriptive statistics were assessed using (SPSS V24, IBM, USA). Data was analyzed for normal distribution by Shapiro Wilk test. Where normal distribution was not present, non-parametric data were stated as median and range. Data were collected and documented anonymously. Missing values were descriptively handled.

### Ethical Approval

The investigation was approved by the local ethics committee of the Hannover Medical School (8172_BO_K_2018). This is a retrospective study and only data were included that were evaluated for patient's treatment.

## Results

### Patient's Characteristics

Forty-four patients (50% women) were included in the analysis with a median age of 63 years (range: 31–84 years) at confirmation of Sjögren's syndrome. Detailed information about the baseline criteria are displayed in Table 1. Female patients presented with a median age of 60 years (range: 45–81 years), and male patients with a median age of 72 years (range: 31–84 years). The median duration between onset of neurological symptoms suggesting peripheral neuropathy and diagnosis of Sjögren's syndrome was 59 months (range 1–231 months).

**Table 1:** Baseline characteristics, ACR/EULAR classification criteria and patient history data.

	**Sjögren's syndrome (included patients)**	**No Sjögren's syndrome (excluded patients)**
Females, *n* (%)	22/44 (50%)	40/140 (59%)
Age (median) at time of Sjögren's syndrome diagnosis		
All patients, years (range)	63 (31–84)	–
Females, years (range)	60 (45–81)	–
Males, years (range)	72 (31–84)	–
ACR/EULAR classification criteria for Sjögren' syndrome		
Evident xerophthalmia, *n* (%)	40/44 (91%)	59/140 (42%)
Evident xerostomia, *n* (%)	18/44 (41%)	31/140 (22%)
Ro-antibody positive, *n* (%)	21/44 (48%)	0/140 (0%)
Sialadenitis with a focus score ≥1, *n* (%)	31/41 (76%)	0/69 (0%)

### Diagnosis of Sjögren's Syndrome

Objective xerophthalmia was identified in all but 4 patients (91%), while 18 patients (41%) showed objective xerostomia. Anti-SSA(Ro) antibodies were present in 21 patients (48%). Minor salivary gland biopsy was performed in all but 3 patients. Sialadenitis with a focus score ≥1 was found in 31/41 patients (76%). The ACR sum score was calculated as 4 in 25/44 patients (57%), as 5 in 11/44 patients (25%), as 7 in 5/44 patients (11%), and as 8 in 3/44 patients (7%).

Serological analysis of parameters outside the ACR-EULAR classification criteria for Sjögren's syndrome (13) revealed an elevated ANA titer (≥1:320) in 23 patients (52%). Rheumatoid factor was found in 7 patients (16%)—in 4 patients combined with anti-SSA(Ro) antibodies. Positive α-Fodrin antibodies occurred in 11 patients (25%)—in 8 combined with antibodies against SSA(Ro). In total, 34 patients (77%) showed either antibodies against anti-SSA(Ro) and/or α-Fodrin, ANA with a titer ≥1:320, or positive rheumatoid factor.

Antibodies against SSA(Ro) were not found in patients who were not diagnosed with Sjögren's syndrome and therefore excluded from further detailed analyses (*n* = 140). Positive α-Fodrin antibodies occurred in 19% of these patients while 4% of patients without Sjögren's syndrome showed positive rheumatoid factor. Additional basic immunological results are shown in Supplementary Table 2.

### Patient's Diagnoses Preceding Diagnosis of Sjögren's Syndrome

**Figure 2:**
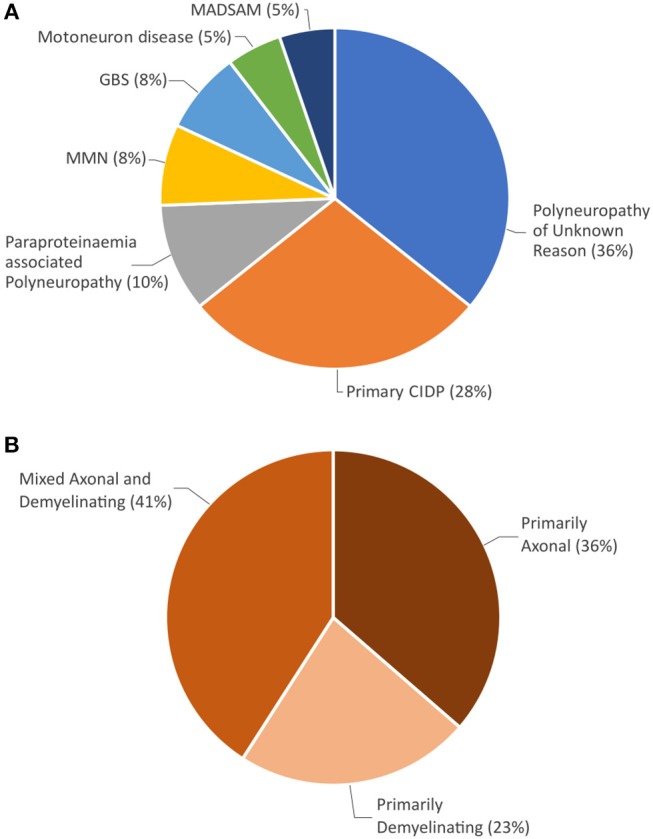
**(A)** Neuropathy diagnoses prior to Sjögren's syndrome diagnosis. **(B)** Distribution of electrophysiological damage pattern at initial neurographic analysis.

Sjögren's syndrome was mostly (41/44; 93%) diagnosed after neurological symptoms had developed. At presentation to our department, 5 patients (11%) received primary workup leading to the diagnosis of peripheral neuropathy and Sjögren's syndrome. The other 39 patients (89%) had previously been diagnosed with neuropathy not associated with Sjögren's syndrome. Preceding diagnoses were polyneuropathy of unknown origin in 14 cases (36%), primary CIDP in 11 cases (28%), paraproteinaemia associated polyneuropathy in 4 cases (10%), multifocal motor neuropathy (MMN) in 3 cases (8%), Guillain Barré Syndrome in 3 cases (8%), motoneuron disease in 2 cases (5%), and multifocal acquired demyelinating sensory and motor neuropathy (MADSAM) in 2 cases (5%) (Figure 2A). Motoneuron disease had been suspected in 5 cases throughout patient's history.

### Patient's Initial Symptoms and Disease Course

Initial symptoms were reported mainly as paraesthesia (17 cases, 39%), weakness (11 cases, 25%), combination of both (6 cases, 14%), and unspecified gait disturbance (5 cases, 11%). Two patients initially presented with muscle cramps (5%) and the remaining 3 patients initially complained about bladder dysfunction, dyspnoea and facial palsy (each *n* = 1). The findings are specified in Table 2. All patients developed weakness of extremities throughout disease course. In addition, cranial nerve involvement occurred in 18 patients (41%) with detailed symptoms described in Table 3. Involuntary muscle contractions were reported in 17 cases (32%). Persistent bladder dysfunction was found in 9 cases (20%).

**Table 2:** Initial symptoms of Sjögren's syndrome.

Paraesthesia (isolated), *n* (%)	17 (39%)
Pareasthesia with paresis, *n* (%)	6 (14%)
Paresis (isolated), *n* (%)	11 (25%)
Gait disturbance, *n* (%)	5 (11%)
Muscle cramps, *n* (%)	2 (5%)
Bladder dysfunction, *n* (%)	1 (2%)
Dyspnoea, *n* (%)	1 (2%)
Facial palsy, *n* (%)	1 (2%)

**Table 3:** Cranial and phrenic nerve impairment.

**Cranial and phrenic nerves affected**	***n* (%)**
Cranial nerve II (anosmia)	1/44 (2.3%)
Cranial nerves III, IV, VI (oculomotor disturbance with diplopia)	2/44 (4.5%)
Cranial nerve V (trigeminal affection with sensory disturbance and neuralgia)	6/44 (13.6%)
Cranial nerve VII (facial nerve palsy)	3/44 (6.8%)
Cranial nerve VIII (hypacusis)	8/44 (18.2%)
Cranial nerves IX, X (dysphagia, hoarseness)	6/44 (13.6%)
Phrenic nerve palsy (dyspnoea, raised diaphragm)	2/44 (4.5%)

Five patients (11%) showed an acute disease course and developed weakness of extremities within days. In those cases, Guillain Barré Syndrome was actively evaluated as initial diagnosis. The remaining patients showed a chronic progressive course of neurological symptoms, while weakness was present at symptom onset in 39% of patients (median 1 month, range 1–130 months).

### Neurological Manifestations at the Time of Worst Documented Clinical Status (Judged by Overall Disability Sum Score)

Weakness of extremities was present in all patients. The most prominent muscle weakness was documented with a median of 3 (by Medical Research Council, range 0–4). Paralysis was distributed symmetrically in 37 patients (84%).

Tetraparesis was the predominant pattern of affected muscles and was found in 25 cases (57%), while 12 patients (27%) suffered from paraparesis. In patients with asymmetric peripheral neuropathy monoparesis occurred in 4 cases (9%) and affection of three extremities was found in 3 cases (7%).

The neurologic examination showed a functional impairment as determined by the overall disability sum score [ODSS] of 3.5 (range 0–12).

Sensory dysfunction was reported by 39 patients (89%). Sensory symptoms occurred symmetrically and showed a stocking- and/ or glove-like distribution in 34 patients (87%). Sensations were reported as pain in 12 cases (31%), numbness or tingle paraesthesia in 35 cases (90%), and dysfunctional proprioception in 32 cases (82%). The full workup is detailed in Table 4.

**Table 4:** Neurologic findings in worst examination (judged by ODSS).

Sensory dysfunction, *n* (%)	39/44 (89%)
Distribution	
Symmetrical, *n* (%)	34/39 (87%)
Stocking-/glove like, *n* (%)	34/39 (87%)
Quality	
Numbness or prickling, *n* (%)	35/39 (90%)
Dysfunctional proprioception, *n* (%)	32/39 (82%)
Pain, *n* (%)	12/39 (31%)
Weakness, *n* (%)	44/44 (100%)
Distribution	
Tetraparesis, *n* (%)	25/44 (57%)
Paraparesis, *n* (%)	12/44 (27%)
Affection of 3 extremities, *n* (%)	3/44 (7%)
Monoparesis, *n* (%)	4/44 (9%)
Most prominent muscle weakness (MRC), median (range)	3 (0–4)
ODSS, median (range)	3.5 (0–12)

### Electrophysiological Findings

Nerve conduction examinations demonstrated impairment of motor nerves in all patients and impairment of sensory nerves in 39 patients (89%) with Sjögren's syndrome.

Nerve damage was classified as predominantly axonal in 16 cases (36%), predominantly demyelinating in 10 cases (23%), and mixed axonal and demyelinating in 18 patients (41%) (Figure 2B).

Evaluation of the electrodiagnostic criteria for a hypothetical diagnosis of CIDP as suggested by the EFNS classification criteria revealed definite CIDP in 23 patients (52%) and probable CIDP in 4 patients (9%) with Sjögren's syndrome.

In addition, patients who were not diagnosed with Sjögren's syndrome and therefore excluded from further analyses (*n* = 140) were evaluated for a hypothetical CIDP. The EFNS criteria suggested definite CIDP in 73 of these patients (52%), probable CIDP in 6 patients (4%), and possible CIDP in 4 patients (3%).

### Cerebrospinal Fluid Findings

**Figure 3:**
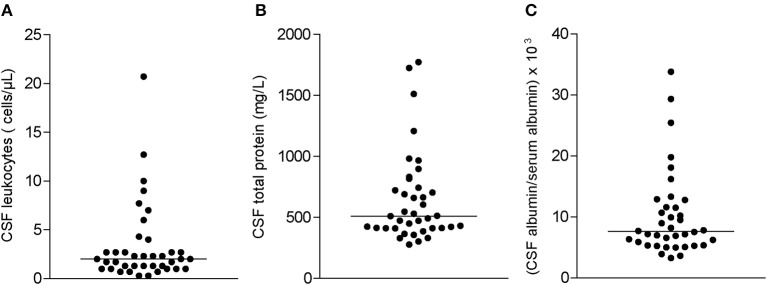
Distribution of cerebrospinal fluid (CSF) parameters in all study patients: **(A)** CSF cell counts, **(B)** CSF total protein, and **(C)** albumin quotient (CSF albumin/serum albumin × 103). Horizontal bars indicate median values.

Inflammatory CSF findings were only rarely found in patients with peripheral neuropathy associated with Sjögren's syndrome. Four patients (9%) showed a slightly elevated cell count of 8, 10, 13, and 21 cells/μl (Figure 3). Total CSF protein was increased in 20 patients (51%). Blood-CSF barrier dysfunction (Qalbumin) was evident in 18 patients (46%). Barrier dysfunction was severe in 3 cases and mild to moderate in 15 cases. All but one patient had normal lactate concentrations. Quantitative intrathecal synthesis of IgG (Reiber graphs) was found in 2 patients (5%). IgM or IgA synthesis did not occur. Oligoclonal bands restricted to the CSF (type 2 and 3) indicating intrathecal IgG synthesis were found in 3 patients (7%).

### Therapeutic Profiles in Patients With Peripheral Neuropathy and Sjögren's Syndrome

Immunomodulatory therapy led to clinical benefit in 97% (32/33) of patients that attended follow-up evaluation (*n* = 33). The remaining 11 patients were lost to follow-up. All patients received multiple therapeutic regimes throughout disease course, resulting in a total of 113 treatment regimes with follow-up evaluation in 81 cases (72%). Changes in therapeutic regime (0–4 changes per patient) were made due to disease progression in 21 cases (31%) and adverse events in 12 cases (18%). Riluzol medication was terminated in 4 cases (6%), after motoneuron disease was no longer suspected. Definite clinical benefit with documented improvement of motor function or stabilization of status quo after previous deterioration was considered a favorable response. Analysis of all therapeutic regimens revealed a favorable response in 66 treatment regimens (81%) (Table 5).

**Table 5:** Therapeutic profiles administered in at least 3 patients.

**Treatments**	**Favorable response**
Treatment regimens with follow up evaluation	66/81
Steroids	
Methylprednisolone (intravenous)	7/8
Prednisolone (oral)	5/10
Primary non-steroid therapy	
Azathioprine	6/6
Azathioprine + intravenous immunoglobulins	2/3
Mycophenolate mofetil	1/3
Intravenous immunoglobulins	22/23
Intensified therapy	
Plasmapheresis	3/3
Cyclophosphamide	3/5
Rituximab	4/4
Rituximab + intravenous immunoglobulins	4/4

## Discussion

This study facilitates the recognition of Sjögren's syndrome as a cause for severe neuropathy with limb weakness, which favorably responds to immunosuppressive therapy. We propose to further refer to Sjögren's associated neuropathy as “Neuro-Sjögren.”

### Severe Neuropathy as an Underestimated Complication of Sjögren's Syndrome

The incidence of neuropathy with fulminant development of limb weakness in Sjögren's syndrome is still unknown. Despite the fact, that several cases have been reported (6, 8, 20), studies including high numbers of patients are not available. In a previous analysis of patients with Neuro-Sjögren presenting to our department between 2004 and 2015, 27 patients with polyneuropathy were described (4). However, 5 patients suffered from neuropathy with limb weakness while the remaining 22 patients complained about sensory deficits only. We therefore systematically investigated all patients presenting with severe polyneuropathy and limb weakness for a possible underlying Sjögren's syndrome. During a time period of 2 years and 5 months 184 eligible patients received tests for xeropthalmia and xerostomia and blood analysis for SSA(Ro) antibodies. In the case of one pathological sign a minor salivary gland biopsy was performed. Sjögren's syndrome could be diagnosed in 44/184 patients (24%). This striking rise of the incidence is explained by the implementation of routine screening for Sjögren's syndrome in our department and suggests that this severe complication is frequently unrecognized.

All 44 patients featured neuropathy with limb weakness in which motor impairment manifested symmetrically in 84% and as tetraparesis in 57%. Sensory function was not affected in 11% of patients indicating that Sjögren's syndrome associated neuropathy can involve solely motor functions. Sensory peripheral neuropathy has been recognized among patients with Sjögren's syndrome with reported rates of peripheral neuropathy between 1.6 and 31% when analyzing patients with Sjögren's syndrome in general (21–25). However, patients with limb weakness were not included or characterization of neuropathy was not performed in these previous cohorts. This might be explained by the fact, that Sjögren's syndrome is not an established diagnosis as a cause of motor impairment in severe polyneuropathy. Patients with sicca symptoms and suspected Sjögren's syndrome are usually referred to rheumatological departments, where diagnosis is reappraised and associated neuropathic complaints are further evaluated. Neuropathy, however, often precedes the development of sicca syndrome (26), rendering the recognition of Sjögren's syndrome during early stages of the disease course nearly impossible. Furthermore, patients presenting with limb weakness as a chief complaint of neuropathy are rather treated in neurological departments, where a potentially causal Sjögren's syndrome is not routinely investigated.

Another contributing factor to the underestimation of Neuro-Sjögren might be the proposition of other causes of neuropathy in neglect of Neuro-Sjögren's diagnosis. Most of our patients had been diagnosed with other entities for a long time, without re-evaluating the validity of the diagnosis in the course of time. Many had received perpetual treatment mainly with intravenous immunoglobulins with short-term clinical benefit and long-term deterioration of symptoms. Some of these patients were thought to suffer from an atypical CIDP. Five patients had even been diagnosed with motoneuron disease until the atypical course prompted re-evaluation of diagnoses.

### Clinical Characteristics of Neuro-Sjögren or “Who to Look Out For?”

The gender distribution showed a balanced male-female ratio of 1:1 in our cohort, whereas Sjögren's syndrome in general has a female predominance between 6:1 and 20:1 (27–29). The discrepancy in gender distribution might result from different patient cohorts analyzed. Most previous studies described patients with Sjögren's syndrome in general and predominantly patients with rheumatic symptoms while our cohort included patients with neuropathy with limb weakness only. However, there are already tendencies in previous reports that show a shift of the gender distribution toward male patients: Sivadasan and colleagues described a cohort of 54 patients with Sjögren's syndrome and neuropathy consisting of only 68.5% females (30), and data from the French ASSESS cohort revealed 88% females in the group with nervous system involvement compared to 95% females in the group without nervous system involvement (31). Other possible reasons for the gender discrepancy might be different pathomechanisms in patients with Sjögren's syndrome and associated organ involvement. Furthermore, progressive paralysis is a major cause for disability and directly affects the quality of life. Thus, the majority of such patients, independent if male or female, undergo a diagnostic work-up, which might be a reason for the balanced male-female distribution. However, additional investigations including larger cohorts of patients are needed to confirm our results. The onset of symptoms was reported at a median age of 63 years and limb weakness was among the initial symptoms in most cases. Limbs were symmetrically involved in 84% of patients and led to a median ODSS of 3.5. Additional sensory symptoms matched the typical signs of polyneuropathy with a mostly stocking- or glove-like-distribution. Symptoms developed rarely within days (11% of patients). In these cases, Guillain Barré syndrome was considered the most likely differential diagnosis and treatment with immunoglobulins was initiated. Intravenous application of immunoglobulins led to temporary improvement of all symptoms, but longterm clinical benefit could not be achieved as symptoms relapsed, and deteriorated over time. In the majority of patients, limb paralysis developed over months indicating a predominantly chronic nature of this disease. Some patients with a pure motor dysfunction were initially misdiagnosed as having a motoneuron disease which aligns with recent case reports about the clinical mimicry of those two entities (6, 32, 33). Those patients responded to immunomodulatory treatment, and thus, the previously suspected diagnosis of motoneuron disease was rejected.

### Diagnostic Tools for Evaluation of Neuro-Sjögren

The ACR-EULAR classification criteria were applied for all patients. Still, not all items were equally represented in our cohort. Objective xerophthalmia was detected in most patients (91%), while objective xerostomia was found in less than half of our cohort (41%). Anti-SSA(Ro)-antibodies were present in only 48% of our patients. The other 52% of patients were diagnosed with Sjögren's syndrome based on sufficient sialadenitis on minor salivary gland biopsy. Antibody status alone is therefore no criterion to rule out Sjögren's syndrome. Furthermore, as some patients frequently received treatments with immunoglobulins before evaluation of antibody status, minor salivary gland biopsy resembles the more reliable diagnostic tool, and should be performed liberally.

Inflammatory CSF findings were rarely found and were therefore not specific for Sjögren-associated polyneuropathy which is in accordance with previous studies (4, 33). Nevertheless, lumbar puncture should be performed to rule out other autoimmune and infectious diseases.

Furthermore, electrophysiological measurements did not reveal pathognomonic findings for Neuro-Sjögren. Every third patient presented with a predominantly axonal damage while every fourth patient showed a predominantly demyelinating damage. The remaining patients revealed signs of both demyelinating and axonal damage. Since many of these patients were investigated in an advanced disease stage the primary pattern remains unclear. Notably, more than half of our patients retrospectively fulfilled the EFNS diagnostic criteria for CIDP. However, similar results concerning a hypothetical CIDP were found in the other 140 patients who did not fulfill the diagnostic criteria for Sjögren's syndrome. These results show that the EFNS criteria are not an appropriate tool for differentiation between CIDP and other immunologically mediated neuropathies such as Neuro-Sjögren. Furthermore, Lozeron et al. recently described patients with transthyretin familial amyloid polyneuropathy who fulfilled the EFNS criteria for a CIDP although these patients did not suffer from an immunologically mediated neuropathy (34).

### Therapeutic Approaches

In our cohort, immunomodulatory therapy led to a clinical benefit with documented improvement of motor function or stabilization of status quo after previous progression in most patients who visited for follow up. Consistent with previous reports we conclude that immunomodulatory therapy is recommendable to ensure clinical benefit for patients with Sjögren-associated polyneuropathy. However, no therapeutic recommendations can be made based on these data due to a low number of patients and since treatment decisions were very heterogeneous. Still there is a vast insecurity concerning the choice of appropriate therapy in the absence of clinical trials focusing on therapy of Sjögren's syndrome associated neuropathy.

### Limitations

All data were collected from patients treated at a university hospital resulting in a selection bias. This is not unusual because most of these patients were treated in other neurological clinics before and were discharged without a sufficient diagnosis and specific treatment. However, the high number of newly diagnosed Sjögren's syndrome in every fourth patient with severe neuropathy with limb weakness might be therefore overestimated. Multicenter studies are required to reach appropriate cohort sizes and minimize selection bias.

### Outlook

During preparation of this manuscript 14 further patients with severe neuropathy with limb weakness have been diagnosed with Sjögren's syndrome indicating that this disorder is more frequent than previously assumed. Raising awareness of Neuro-Sjögren is therefore crucial—especially in patients with severe peripheral neuropathy.

Defining a stereotype patient for Neuro-Sjögren is challenging as patient characteristics in terms of comorbidities and severity of symptoms were heterogenous in our cohort. Some patients had been treated with intravenous immunoglobulins and had improved temporarily. Hence, further diagnostic steps were neglected, even when symptoms relapsed, and instead the diagnosis of an atypical CIDP was suggested. In this scenario, the dosage of immunoglobulins was often increased which again improved patients' symptoms temporarily. Some of the older patients were not treated with immunomodulatory substances as age-associated and therefore unchangeable causes for evident neuropathy were implied. In severe progressive motor dysfunction with permanent dependence to wheelchair or confinement to bed phrenic nerve paralysis and dysphagia occurred in some patients, and thus, a motoneuron disease was suspected. These patients improved after implementation of immunomodulatory/immunosuppressive treatment. It is therefore crucial to evaluate a wide variety of patients for Neuro-Sjögren even if symptoms seem to be of fatal dimension.

## Conclusion

Our data indicate that severe neuropathy with limb weakness is often associated with Sjögren's syndrome. However, there are no pathognomonic clinical signs or electrophysiological findings and therefore distinction between Sjögren' syndrome and other causes of neuropathy such as CIDP can be challenging, especially in early disease stages. We thus suggest performing screening tests for Sjögren's syndrome in all patients with polyneuropathy and especially in those with motor dysfunction. As neurologic manifestations frequently precede sicca symptoms and laboratory findings (i.e., anti-SSA(Ro)-antibodies), perpetual re-evaluation for objective xerophthalmia or xerostomia should be performed. In case of pathological tear or saliva production, minor salivary gland biopsy should be performed in order to diagnose a potential Sjögren's syndrome in time.

## Ethics Statement

This study was approved by the institutional ethics committee of the Hannover Medical School (8172_BO_K_2018). This is a retrospective study and only data were included that were evaluated for patient's treatment. Thus, the local ethics committee waived the need for written informed consent from the participants.

## Author Contributions

Recruitment of patients and processing of patient history data were accomplished by TaS, LB, TT, SG, and K-WS. NP contributed in testing for xerophthalmia and significantly drafting the manuscript. BS drafted a substantial portion of the manuscript. Nerve conduction studies and their interpretation were obtained by SK. TW, MS, and ThS provided expertise for conception and design of the study. All authors contributed to manuscript revision, read, and approved the submitted version.

### Conflict of Interest Statement

The authors declare that the research was conducted in the absence of any commercial or financial relationships that could be construed as a potential conflict of interest.

## Supplementary Material

Supplementary Table 1 Applied electrophysiological classification criteria for nerve damage patterns.

Supplementary Table 2 Extended immunological serologic parameters.
